# Differential expression of the capsaicin receptor TRPV1 and related novel receptors TRPV3, TRPV4 and TRPM8 in normal human tissues and changes in traumatic and diabetic neuropathy

**DOI:** 10.1186/1471-2377-7-11

**Published:** 2007-05-23

**Authors:** Paul Facer, Maria A Casula, Graham D Smith, Christopher D Benham, Iain P Chessell, Chas Bountra, Marco Sinisi, Rolfe Birch, Praveen Anand

**Affiliations:** 1 Peripheral Neuropathy Unit, Imperial College, Hammersmith Hospital, London, UK; 2 Neurology and Gastrointestinal Diseases Centre of Excellence for Drug Discovery, GlaxoSmithKline, Harlow, UK; 3 Peripheral Nerve Injury Unit, Royal National Orthopaedic Hospital, Stanmore, UK

## Abstract

**Background:**

Transient receptor potential (TRP) receptors expressed by primary sensory neurons mediate thermosensitivity, and may play a role in sensory pathophysiology. We previously reported that human dorsal root ganglion (DRG) sensory neurons co-expressed TRPV1 and TRPV3, and that these were increased in injured human DRG. Related receptors TRPV4, activated by warmth and eicosanoids, and TRPM8, activated by cool and menthol, have been characterised in pre-clinical models. However, the role of TRPs in common clinical sensory neuropathies needs to be established.

**Methods:**

We have studied TRPV1, TRPV3, TRPV4, and TRPM8 in nerves (n = 14) and skin from patients with nerve injury, avulsed dorsal root ganglia (DRG) (n = 11), injured spinal nerve roots (n = 9), diabetic neuropathy skin (n = 8), non-diabetic neuropathic nerve biopsies (n = 6), their respective control tissues, and human post mortem spinal cord, using immunohistological methods.

**Results:**

TRPV1 and TRPV3 were significantly increased in injured brachial plexus nerves, and TRPV1 in hypersensitive skin after nerve repair, whilst TRPV4 was unchanged. TRPM8 was detected in a few medium diameter DRG neurons, and was unchanged in DRG after avulsion injury, but was reduced in axons and myelin in injured nerves. In diabetic neuropathy skin, TRPV1 expressing sub- and intra-epidermal fibres were decreased, as was expression in surviving fibres. TRPV1 was also decreased in non-diabetic neuropathic nerves. Immunoreactivity for TRPV3 was detected in basal keratinocytes, with a significant decrease of TRPV3 in diabetic skin. TRPV1-immunoreactive nerves were present in injured dorsal spinal roots and dorsal horn of control spinal cord, but not in ventral roots, while TRPV3 and TRPV4 were detected in spinal cord motor neurons.

**Conclusion:**

The accumulation of TRPV1 and TRPV3 in peripheral nerves after injury, in spared axons, matches our previously reported changes in avulsed DRG. Reduction of TRPV1 levels in nerve fibres in diabetic neuropathy skin may result from the known decrease of nerve growth factor (NGF) levels. The role of TRPs in keratinocytes is unknown, but a relationship to changes in NGF levels, which is produced by keratinocytes, deserves investigation. TRPV1 represents a more selective therapeutic target than other TRPs for pain and hypersensitivity, particularly in post-traumatic neuropathy.

## Background

The cloning of the vanilloid receptor-1 (TRPV1) [1,2] has led to greater understanding of the mechanisms of thermosensation and the effects of capsaicin, the noxious component from chilli peppers. TRPV1 is a non-selective, cation channel activated by capsaicin and heat (42°C or greater), and is a member of the transient receptor potential (TRP) family of temperature sensitive ion channels. Thermal sensations and pain are detected via sub-sets of neurons which are activated within distinct temperature ranges, from cool (<25°C – 28°C – TRPM8 [3]), warm (>27°C – 38°C – TRPV3[4,5] and TRPV4 [6,7] to noxious/painful heat (>43°C – 52°C – TRPV2 [8] and TRPV1[1,2]) and cold (<17°C -TRPA1) sensations[3,9,10].

Studies of TRPV1 in animal models have revealed its role in heat and pain mechanisms [11,12]. Subsequent studies indicated that it may not act as the only receptor for heat [13] especially since the response of neurons to heat and capsaicin are not always identical [14]. Searches of the GenBank nucleotide databank revealed an unfinished human sequence homologous to TRPV1, which has since been identified as a temperature-sensitive but capsaicin and pH insensitive, vanilloid receptor-like protein nominated as TRPV3 [4,5]. Although vanilloid receptors are known to exist and function as homomers [15,16], some evidence has been provided for the biochemical association of TRPV3 and TRPV1 suggesting heteromerization [17], thus allowing a greater range of receptor characteristics. In addition to its co-localization with TRPV1 in small/medium diameter sensory neurons of the dorsal root ganglion (DRG), the number of both TRPV3- and TRPV1- immunoreactive sensory neurons increased significantly after DRG avulsion injury i.e. central axotomy [4]. In genetically modified mice lacking the TRPV1 receptor, thermal hyperalgesia was impaired [18]. In animal models of nerve injury, TRPV1 mRNA was reported to be down-regulated after axotomy [19] but up-regulated in spared nerve fibres [20]. Other studies have demonstrated changes in the molecular phenotype of undamaged neurons in neuropathic pain models of nerve ligation. In the Seltzer model, where undamaged afferents may be identified by retrograde labelling, the expression of the neuropeptides substance P and galanin, both known nociceptive mediators, as well as mRNA for the sodium channel SNS, increased in the somata of undamaged fibres [21-23].

Relatively little is known of vanilloid receptors in human nerve injury and skin, and their relationship to pain or hypersensitivity. Nerve injury-induced alterations of sodium channel density and distribution is thought to contribute to pain by generation of ectopic discharges from the neuroma or DRG [24]. Previous studies of injured human nerves have shown that some sodium channel subunits accumulate in proximal nerve stumps [25] and neuromata [26] while others can be switched on [24] thus contributing to changes in membrane excitability and/or pain. Peripheral nerve injury in humans may lead to changes in skin sensitivity depending upon the level at which injury is sustained and its severity. Numbness, hypo- or hyper-algesia and allodynia are common symptoms, sometimes in combination, which may arise over many years following injury or surgical repair. Such variability in sensation is due to processes of nerve regeneration and re-innervation of the skin, and may lead to such phenomena as paradoxical sensation (burning sensation on cooling the skin). Phenotypic change of primary afferents with respect to expression of TRPs may be one possible explanation for some of these symptoms and signs.

TRPV3 and TRPV1 are present not only in DRG sensory neurons but also in various regions of the central nervous system and non-neuronal tissue [5,27]. TRPV3, for example, has been detected in rodent keratinocytes [28]. In addition, TRPV1-immunoreactivity has been shown to be present in cultured keratinocytes where its activation by capsaicin induces the production of pro-inflammatory mediators such as COX-2, IL-8 and PGE-2 [29,30]. The presence of vanilloid receptors in keratinocytes thus provides a potential for keratinocyte/nerve interaction [28], but these findings and their physiological relevance remain controversial. Basal and supra-basal keratinocytes also produce the neurotrophins NGF and NT-3 respectively [31,32] and TRPV1 is known to be regulated by neurotrophins, i.e. NGF and GDNF [33-38]. Evaluation of epidermal innervation has proven to be helpful for the assessment of diabetic and other small fibre neuropathies, where innervation has been related to sensory changes [31,39-43]. In an animal model of diabetes, thermal allodynia and hyperalgesia were associated with sensitisation of TRPV1 receptors in spinal cord [44].

TRPV4 is a nonselective cation channel, first described as an osmosensor [6], whose opening seems to result from tyrosine kinase-dependent phosphorylation [5]. It is activated at temperatures above 27°C and expressed and functions as an osmosensor in rodent nociceptors [45]. Two further, temperature sensitive, ion channels TRPM8 and TRPA1 are localised in sensory neurons and are sensitive to cool/cold temperatures < 25–28°C for TRPM8 and < 17°C for TRPA1 [3,5,9].

The aim of the present study was to investigate the distribution of the vanilloid receptors TRPV1, TRPV3, TRPV4, and TRPM8 in normal and injured human peripheral nerves and spinal nerve roots and in normal and neuropathic (painful neuroma and diabetes) skin and spinal cord,

## Methods

### Tissues

Specimens of injured nerve (proximal to site of injury) and dorsal root ganglion (DRG) were obtained during surgery for brachial plexus repair. Specimens were sub-divided according to the delay between date of injury and tissue collection at surgery, where acute was defined as less than 3 weeks delay (acute nerves: n = 6; male 5; age range 14 – 34 y; chronic: n = 8; male 5; age range 15 – 43 y; and acute DRG: n = 5; male 4; age range 18 – 50 y; chronic DRG: n = 6; male 6; age range 18 – 30 y). Control nerves (n = 8; male 6; age range 36 – 81 y) were obtained during routine surgery for limb amputation or similar procedures, and control DRG (n = 8; male 3; age range 41 – 98 yr) obtained via the Netherlands Brain Bank with less than 12 h post-mortem delay. Dorsal and ventral pairs of injured (root avulsion) spinal nerve roots were obtained also during brachial plexus repair (n = 9, 4 acute and 5 chronic; male 8; age range 22 – 47 y). Neuropathic, sural nerve biopsies (n = 6; 4 male; age range 49 – 71 yr) were obtained from patients with neuropathies, including demyelinating and polyneuropathies. Control, human spinal cords (n = 2; male 1; age 60 and 64 y), with small attached nerve roots, were obtained after less than 12 h post-mortem delay via the Netherlands Brain Bank. Normal, hyper- and hypo-sensitive skin and painful neuroma samples were collected from the amputated arm of a patient who had a brachial plexus injury with subsequent repair 8 years previously (n = 1; male, age 46 y);. Full thickness, lateral calf skin biopsies were obtained from diabetic patients (n = 8; male 8; age range 36–65 y) under local anaesthetic, and control calf skin biopsies were obtained from patients undergoing harvest of intact sural nerve for brachial plexus repair, or therapeutic elective limb amputation for a non-neurological condition (n = 8; male 5; age range 33–71 y). Skin samples were obtained from excised, supernumerary human digits (n = 2). Fully informed consent was obtained for all tissues collected, with approval of the Local Ethics Committees.

### Immunocytochemistry

Tissues were snap frozen in liquid nitrogen and stored at -80°C until use. For cryomicrotomy, nerves were orientated longitudinally and spinal cord transversally, while skin specimens were orientated to optimise perpendicular dermal papillae. Frozen, unfixed sections (10 μm) were collected onto poly-L-lysine-coated (Sigma Poole Dorset UK) glass slides and post-fixed in freshly prepared, 4% w/v paraformaldehyde in PBS (0.1 M phosphate; 0.9% w/v saline; pH 7.3). After washing in PBS, endogenous peroxidase was blocked by incubation with 0.3% w/v hydrogen peroxide in methanol. After a further wash in PBS, the tissue sections were incubated overnight with primary antibodies (Table 1) including antibodies to the novel human TRPV4 raised against the N-terminal amino acids MADSSEGPRAGPGEVA(C). For double staining, TRPV1 or TRPV3 antibodies were mixed with the neuronal marker peripherin (PPN). For co-localisation of TRPV4 and TRPV1 or TRPV4 and TRPV3, pairs of serial, "mirror image" sections, each containing parts of the same cells, were used for immunostaining. Method controls included omission of primary antibodies or their replacement with pre-immune serum. Specificity controls included pre-incubation of primary antibodies with homologous antigen at 10 ^-1 ^to 10 ^-6 ^mg per ml of diluted antibodies prior to immunostaining. Specificity of antibodies to TRPV1 and TRPV3 has been described in a previous publication [4]. Sites of antibody attachment were revealed using a nickel enhanced ABC (peroxidase; Vector Laboratories, High Wycombe, Bucks., U.K.) method [46].

**Table 1:** Antibody Characteristics

**Antibody**	**Host**	**Source**	**Titre**
Human TRPV3	Rabbit	GSK, Harlow, UK.	1:1000
Human TRPV1	Rabbit	GSK, Harlow, UK.	1:10000
Human TRPV4	Rabbit	GSK, Harlow, UK.	1:1000
TRPM8	Rabbit	GSK, Harlow, UK.	1:1000
TRPM8	Rabbit	Phoenix Pharmaceuticals, Belmont, CA, USA	1: 250
Peripherin	Mouse	Novocastra, Newcastle, UK	1:500
Neurofilament	Mouse	Dako Cytomation, Cambs. UK	1:10000

TRPV: Transient Receptor Potential Vanilloid; TRPM: Transient Receptor Potential Melastatin; GSK: GlaxoSmithKline; Titre represents final working dilution of primary antibodies.

### Analysis

Computerized image analysis was used (Seescan Cambridge, UK) to quantify immunoreactive nerve fibres. Images were captured via video link to an Olympus BX50 microscope (x20 objective) and scanned by the computer. Three to five fields from two tissue sections were analysed where positive immunostaining was highlighted by setting grey-level detection limits at threshold and the area of highlighted fibres obtained as percentage area of the field scanned.

The size (small/medium or large diameter) and number of nucleated sensory neurons for each DRG section were assessed using a calibrated microscope eyepiece graticule and the number of positive TRPV4 cells expressed as % of total neurons for each DRG sample.

The intensity of immunoreaction for TRPV3 or TRPV4 in basal keratinocytes was graded by two independent observers on an arbitrary scale from negative (0) to maximum (3). TRPV1-, neurofilament- and PPN- immunoreactive sub-epidermal fibres were counted for each section and the length of epidermis measured using a microscope eyepiece graticule.

All statistical analysis used non-parametric Mann-Whitney t test.

## Results

A summary of results obtained in the tissues examined are presented in tabular form (Table 2).

### DRG

**Table 2:** Results Summary

	**TRPV1**	**TRPV3**	**TRPV4**	**TRPM8**
**Nerve injury**	Increased (brachial plexus)	Increased (brachial plexus)	Positive Unchanged	Positive. Reduced
**Hypersensitive skin**	Increased	Not detected	Fibres Unchanged	Positive Increased?
**DRG injury**	Decreased (see Smith et al 2002)	Decreased (see Smith et al 2002)	Positive Unchanged Co-localise with TRPV1/TRPV3	Positive Unchanged
**Diabetic skin**	Decreased fibres Negative keratinocytes	Nerve fibres not detected Trend for decreased keratinocytes	Positive fibres – very few	Positive Unchanged
**Neuropathic nerve**	Positive fibres Decreased	None detected	Not examined	Positive Unchanged
**Spinal cord**	Positive dorsal horn	Positive motor neurones	Positive weak motor neurones	Weak fibres in dorsal horn and roots
**Dorsal roots**	Positive fibres	Not detected	Positive fibres	Positive fibres
**Ventral roots**	Not detected	Positive fibres	Positive fibres	Positive fibres

TRPV: Transient Receptor Potential Vanilloid; TRPM: Transient Receptor Potential Melastatin; DRG: Dorsal Root Ganglion.

**Figure 1:**
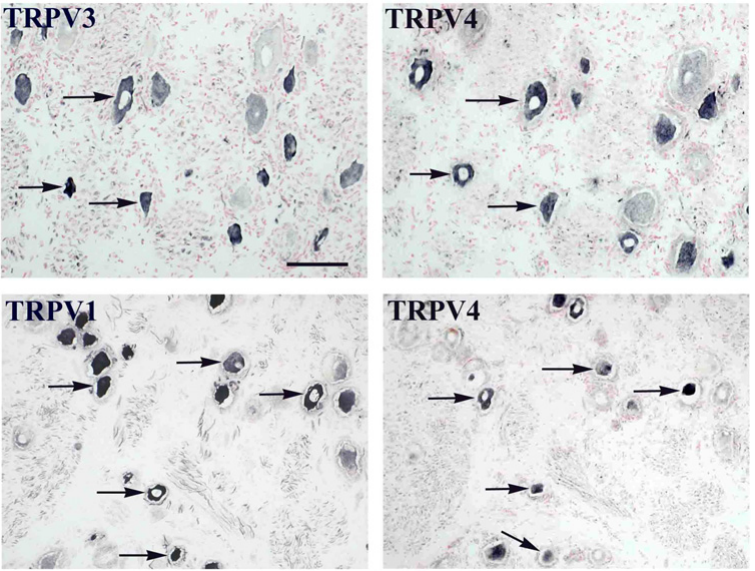
**Co-localisation of TRPV4 with other TRPV receptors in DRG**. TRPV3 and TRPV1 immunoreactivity mostly co-localise with TRPV4 in small/medium neurons. Arrows indicate the same cells in serial sections. Scale bar = 75 μm.

Antibodies to TRPV4 reacted strongly with small/medium but weak with large diameter neurons (Fig 1) with no significant change after injury [% TRPV4 small/medium cells, median (range): controls 65.5 (57–73); acute 54 (48–68); chronic 60 (47–75); large cells: controls 63 (22–84); acute 56 (56–59); chronic 57 (44–78)]. Co-localisation (serial 'mirror-image' sections) revealed that most TRPV4 positive, small/medium cells were also TRPV1 and TRPV3 positive (Fig 1). Pre-incubation of primary antibodies to TRPV4 with homologous peptide antigen completely prevented staining at 10^-2 ^mg/ml diluted antibodies.

**Figure 2:**
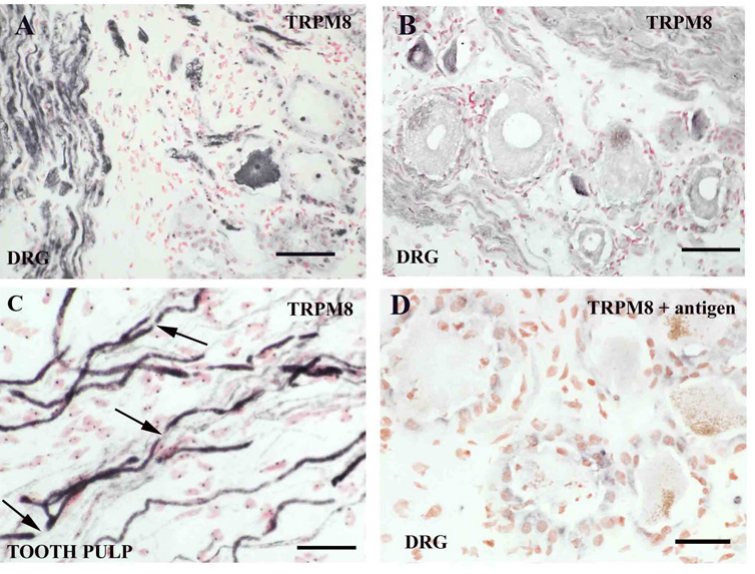
**TRPM8 in DRG and fibres**. TRPM8 immunoreactivity in small/medium diameter neurons using antibodies from GSK (**A**), or Phoenix Pharmaceuticals (**B**) and in fibres in tooth pulp with gaps indicating nodes of Ranvier (**C -**arrows). Pre-incubation of antibodies with antigen gave no staining (**D**). Scale bar = 50 μm.

Strong TRPM8-immunoreactive fibres were detected in all DRG although sensory cell bodies (small/medium diameter) were few but detected using antibodies from two independent sources (Fig 2A,B). There was no obvious change of this pattern after chronic or acute DRG avulsion injury. In samples of human tooth pulp, which contain C and A delta sensory fibres, TRPM8-immunoreactivity was seen in large calibre, fibres (Fig 2C), with identifiable narrow gaps indicative of nodes of Ranvier (Fig 2C arrows), and in fine calibre fibres. Pre-incubation of TRPM8 antibodies (GSK 1323) with homologous antigen abolished staining (Fig 2D).

### Peripheral nerve

**Figure 3:**
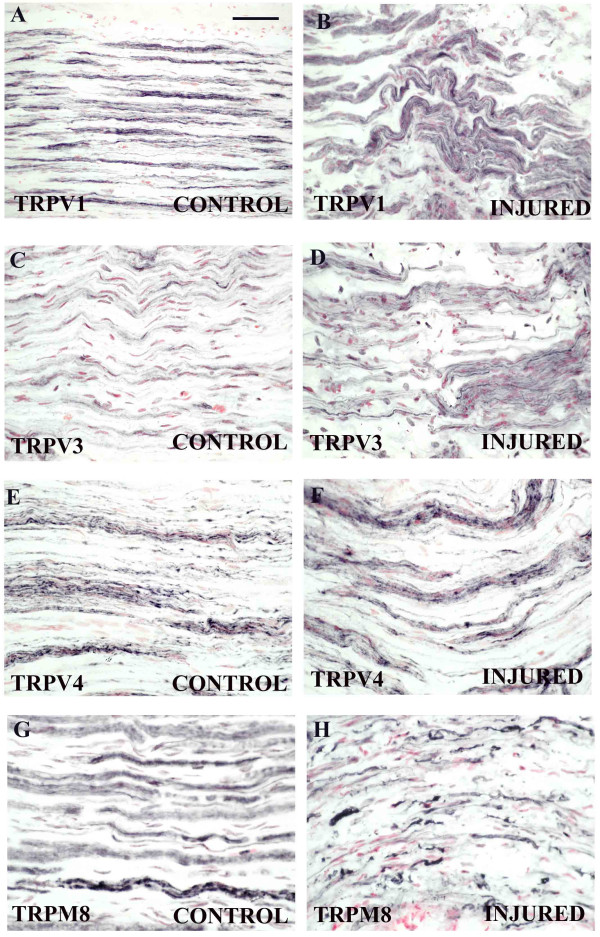
**TRP receptors in peripheral nerve**. TRPV1 (**A**, **B**), TRPV3 (**C**, **D**), TRPV4 (**E**, **F**) and TRPM8 (**G**, **H**) immunoreactivity in subsets of fibres in control, uninjured and injured peripheral nerves. Scale bar = 75 μm.

In control, uninjured peripheral nerve, immunoreactivity for TRPV1 (Fig 3A), TRPV3 (Fig 3C), and TRPV4 (Fig. 3E) was detected in fine to medium calibre fibres with TRPV3 immunoreactivity less intense than that for TRPV1 or TRPV4. After nerve injury both TRPV3 (Fig. 3D) and TRPV1 (Fig 3B) – immunoreactive fibres appeared to be increased in both number and intensity but there was no change for TRPV4 (Fig 3F).

**Figure 4:**
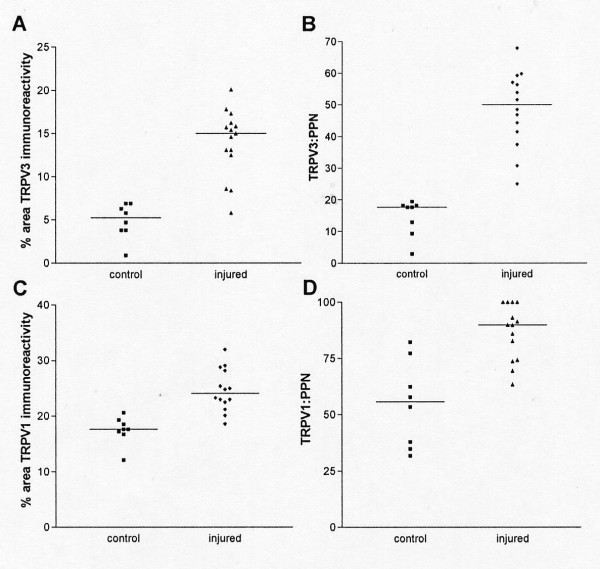
**TRPV3 and TRPV1 in peripheral nerves**. Area (%) of TRPV3 (**A**) and TRPV1 (**C**) in control and injured nerves and comparison (ratio) with total peripherin (PPN) nerve area (%) for TRPV3 (**B**) and TRPV1 (**D**).

Peripheral nerves also displayed immunoreactivity for TRPM8 which was strong in large calibre fibres and emphasized nodes of Ranvier as described above thus suggesting an association or cross reaction with myelin in glial/Schwann cells (Fig 3G). After nerve injury and particularly distal to injury, TRPM8-immunoreactivity in nerve fibres was deterioriated and fragmented further suggesting its presence in glial/Schwann cells (Fig 3H). Immunoreactivities for both TRPV3 and TRPV1 (% area immunoreactive nerve) were significantly increased after injury [median (range) TRPV3: control 5.25 (0.9–6.9), injured 15.0 (5.8–20.1) p < 0.001; TRPV1; control 17.6 (12.1–20.6), injured 24.1 (18.6–32.0) p < 0.001; Fig 4A,C] and although image analysis of the nerve marker peripherin showed no change, % ratios of TRPV3 or TRPV1 to peripherin were significantly increased also [median (range) % TRPV3:PPN, control 17.6 (2.9–19.4), injured 50.1 (25.0–67.9), p < 0.001; % TRPV1:PPN, control 55.7 (31.9–82.3), injured 89.8 (63.4–100.0), p < 0.001; Fig 4B,D].

**Figure 5:**
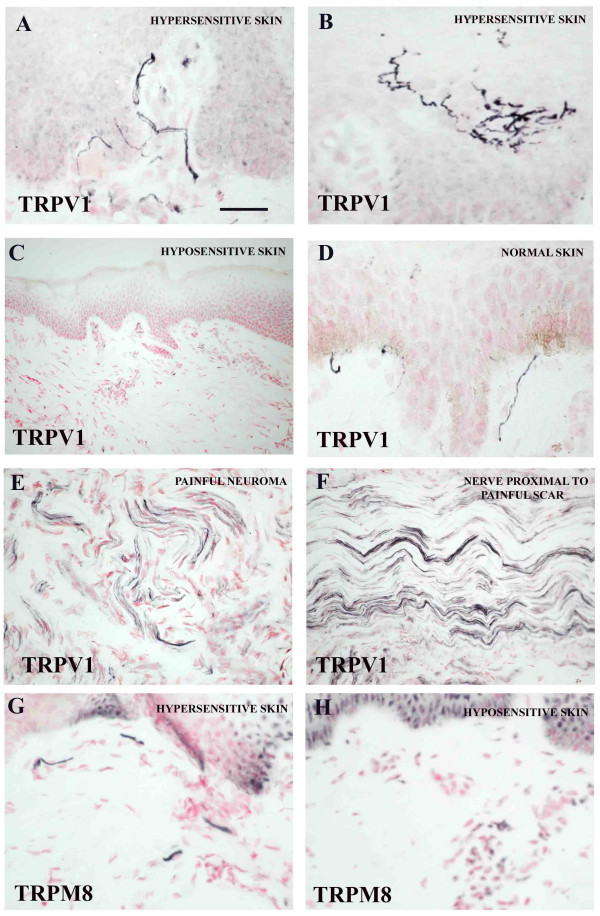
**TRPV1 in hypersensitive skin**. TRPV1-immunoreactive fibres in: thenar eminence (**A**, **B**) from a patient with partial damage to C6 and complete avulsion of C7-T1 and hypo-sensitive skin (**C**) from the ulnar border of his distal forearm; normal skin (**D**); painful peripheral neuroma (**E**) and nerve proximal to an area of painful scar neuritis (**F**); TRPM8-immunoreactive fibres in the sub-epidermis of hypersensitive (**G**) but not in hyposensitive skin (**H**). Scale bar = 25 μm A, B, D, G, H; 50 μm E, F; 100 μm C.

In tissues collected from a subject at limb amputation, with partial damage at C6 root and complete avulsion at C7-T1, with subsequent plexus repair, abundant TRPV1 immunoreactive, sub-epidermal fibres were present in hypersensitive skin (Fig 5A,B) but very few in an adjacent hyposensitive skin region (Fig 5C). In comparison, normal sensate skin (Fig 5D) showed fewer TRPV1-immunoreactive sub-epidermal fibres than hypersensitive skin. TRPV1-immunoreactive fibres were detected in a painful peripheral neuroma (Fig 5E) and in a nerve proximal to an area of painful scar neuritis (Fig 5F). Frequent, TRPM8-immunoreactive large calibre fibres were detected in the sub-epidermal region of hypersensitive (Fig 5G) but not hyposensitive (Fig 5H) skin. In these same tissue samples, TRPV3 immunostaining of peripheral fibres was below detection level.

### Nerve roots and spinal cord

**Figure 6:**
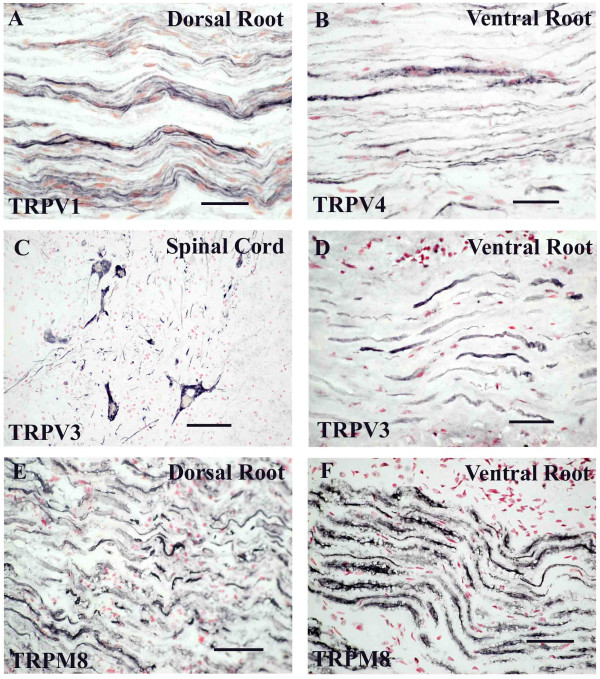
**TRP receptors in spinal cord and roots**. TRPV1 (**A**) in dorsal and TRPV4 (**B**) in ventral spinal roots. Strong TRPV3-immunoreactivity in motor neurons (**C**) and ventral roots (**D**). TRPM8-immunoreactive fibres in dorsal (**E**) and ventral (**F**) spinal roots. Scale bar = 50 μm.

Dorsal but not ventral injured spinal roots, and dorsal roots attached to post-mortem spinal cord, showed strong TRPV1 immunoreactivity (Fig 6A). No TRPV1-immunoreactivity was detected in motor neurons or in the ventral horn of the spinal cord.

TRPV4 was present in injured roots, both dorsal and ventral (Fig 6B), but was weak in motor neurons and fibres in the ventral horns. There was no significant staining in the dorsal horns.

Four out of 9 samples showed strong TRPV3-immunoreactivity in motor neurones and fibres in the ventral horn of the spinal cord (Fig 6C). In these positive samples, counts of TRPV3 motor neurones were 33% of total. In accord, there was strong TRPV3 immunoreactivity, mostly in large calibre fibres, in ventral (Fig 6D) but not dorsal spinal roots. TRPV3 immunoreactivity in the ventral roots was largely lost three to four weeks following injury.

Antibodies to TRPM8 showed fibres in both dorsal (Fig 6E) and ventral (Fig 6F) spinal roots.

### Neuropathic nerves

**Figure 7:**
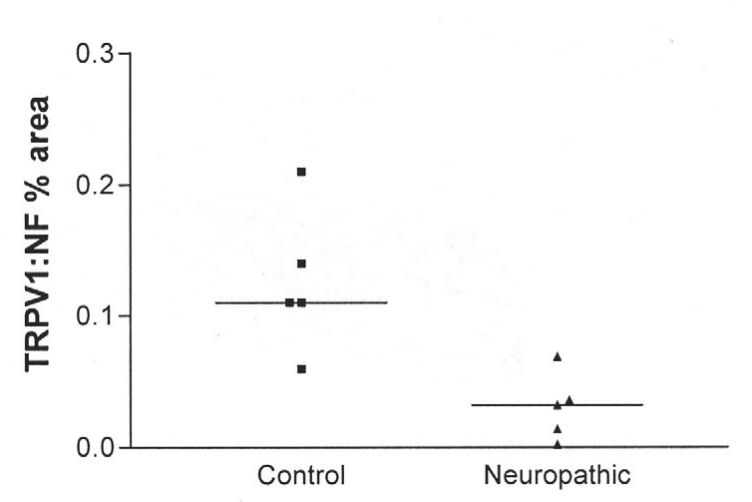
**Non-diabetic neuropathic nerves**. The ratio TRPV1: neurofilament (NF) for image analysis values (% area) is significantly decreased for neuropathic nerves (p < 0.02).

Non-diabetic neuropathic peripheral nerves showed strong immunostaining for neurofilaments, with a sub-population of strong fibres immunoreactive for TRPV1. Image analysis of TRPV1 and the nerve marker neurofilaments in serial sections showed that the ratio of immunoreactive area (% area) for TRPV1: neurofilaments was significantly decreased in virtually all neuropathic nerves regardless of pathology [median (range); control 0.11 (0.06–0.21); neuropathic 0.032 (0.002–0.06); p < 0.02; Fig 7]. There was no obvious change of TRPM8 immunoreactivity.

### Diabetic neuropathy skin

TRPV1-immunoreactive fibres were detected in distal peripheral nerve and were present in fine fibres in control skin up to and including the epidermis, whilst TRPV3-immunoreactivity in fibres was relatively weak in the nerve trunk and undetectable at the skin level but was present in basal keratinocytes (see below).

**Figure 8:**
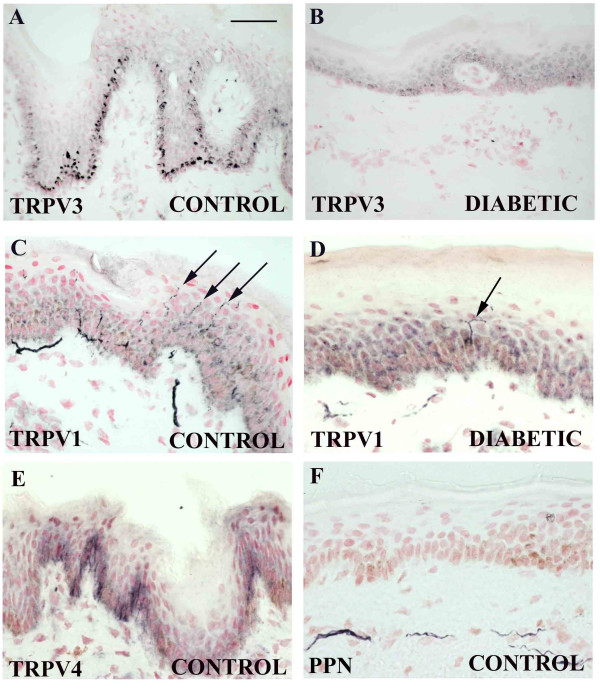
**TRPV3 and TRPV1 immunoreactivity in diabetic skin**. TRPV3-immunoreactive basal keratinocytes in control (**A**) and diabetic (**B**) skin. TRPV1-immunoreactive dermal and epidermal (arrows in C) fibers in control (**C**) and diabetic (**D**) skin. TRPV4 – immunoreactivity in keratinocytes (**E**) and nerve marker (peripherin PPN) in control skin (**F**). Scale bar = 50 μm A, B, D, E ; 25 μm C, F.

TRPV3 immunoreactivity was detected most strongly in basal keratinocytes in normal skin, sometimes continuously along the length of the epidermis (Fig 8A) whilst diabetic skin showed weaker cells (Fig 8B). TRPV3 immunoreactivity was not detected in fibres in any sample. In contrast, TRPV1 immunoreactivity was present in fibres throughout the dermis with fine fibres penetrating the epidermis of control (Fig 8C – arrows) and fewer in diabetic skin (Fig 8D). Keratinocytes showed little or no immunoreactivity for TRPV1.

Few TRPV4-immunopositive nerve fibres were detected in skin samples. Positive but patchy immunostaining of TRPV4 basal keratinocytes was observed (Fig 8E). Sub-epidermal fibres were detected using antibodies to peripherin (PPN; Fig 8F)

As described above, antibodies to TRPM8 showed strong large calibre and weak fine calibre fibre bundles up to the sub-epidermal layer. TRPM8 immunoreactivity was not detected in keratinocytes.

**Figure 9:**
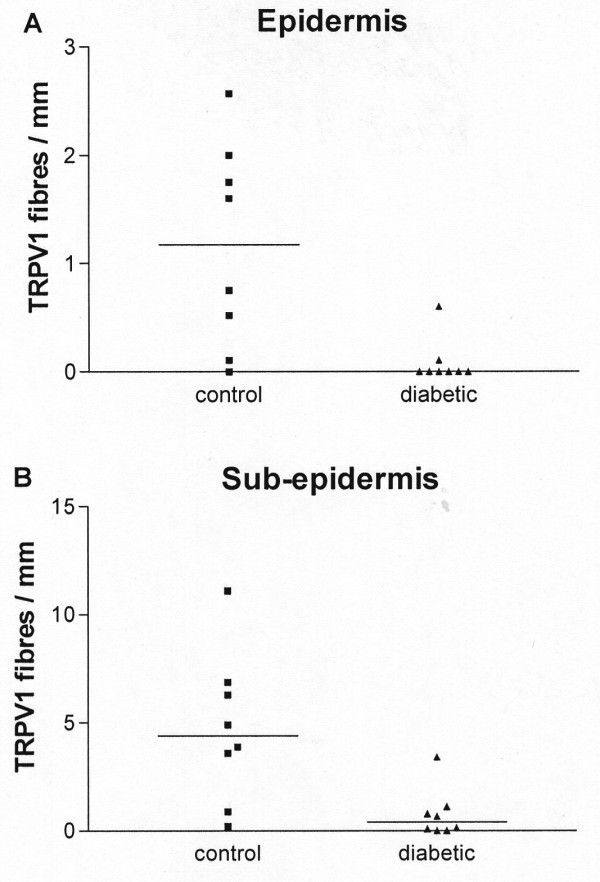
**Quantification of TRPV1-immunoreactive fibres in skin**. Counts of epidermal (**A**) and sub-epidermal (**B**) fibres were significantly reduced (p < 0.05) in diabetic skin.

**Figure 10:**
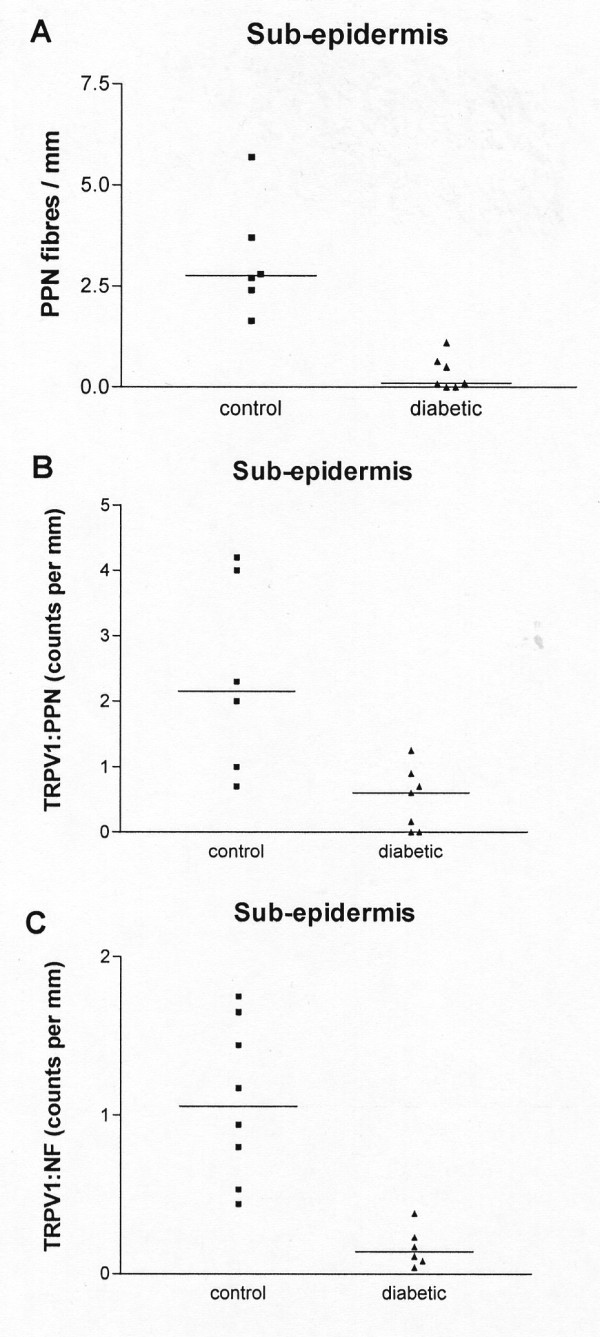
**Quantification of nerve fibres in sub-epidermis**. Peripherin (PPN) – immunoreactive nerve fibres per mm length of skin section (**A**) and ratios of TRPV1: PPN (**B**) or TRPV1: NF (**C**) are significantly reduced in diabetic skin.

In diabetes, counts of TRPV1 immunoreactive fibres per mm length of tissue section showed a significant decrease in both epidermis and sub-epidermis [median (range) Epidermis: control 1.2 (0.0–2.6), diabetic 0.0 (0.0–0.6); p < 0.01; Sub-epidermis: control 4.4 (0.2–11.1), diabetic 0.4 (0.0–3.4); p < 0.01; Fig 9A,B]. In order to determine whether this decrease of TRPV1-expressing fibres is due solely to the denervation associated with diabetes, the number of TRPV1-immunoreactive fibres was correlated with the number of fibres obtained with the neuronal markers neurofilaments or peripherin. In the sub-epidermis, peripherin- but not neurofilament -immunoreactive fibres were significantly reduced [PPN fibres per mm, median (range) control 2.8(1.6–5.7), diabetic 0.1(0.0–1.1); p < 0.01; Fig 10A]. Comparison of the ratio of TRPV1 with either neuronal marker showed a significant decrease in diabetic sub-epidermis [TRPV1: peripherin median (range): controls 2.2 (0.7–4.2); diabetic 0.6 (0–1.3) p < 0.01; TRPV1: neurofilament median (range): controls 1.05 (0.44–1.75); diabetic 0.14 (0.04–0.38) p < 0.01; Fig 10B,C] indicating a decrease of TRPV1 immunoreactivity preceding the decrease in innervation.

**Figure 11:**
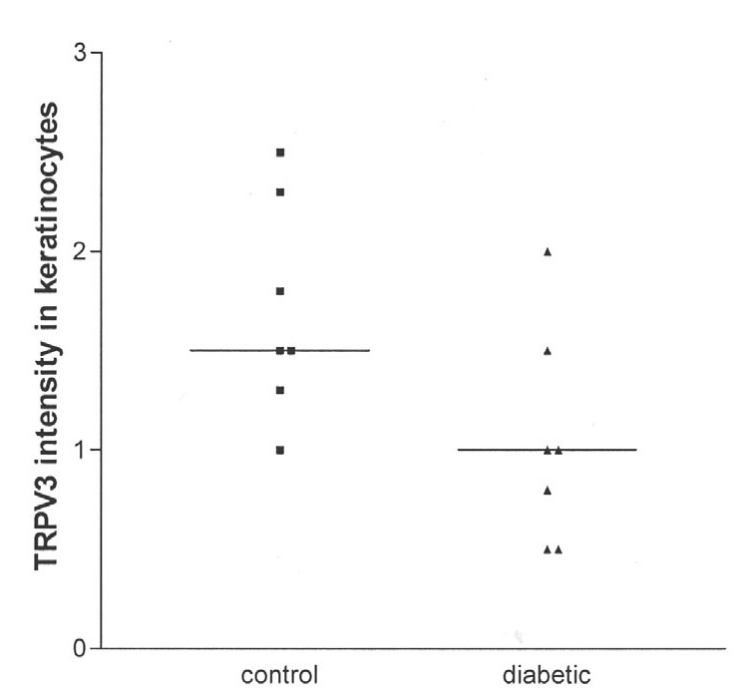
**TRPV3 immunostaining in keratinocytes**. The intensity of TRPV3 immunostaining in keratinocytes is decreased in diabetic skin compared to control. The median value is indicated.

Visual assessment of the intensity of TRPV3 and TRPV4 immunostaining in keratinocytes in diabetic skin showed a trend for decrease of TRPV3 [median (range) control 1.5 (1.0–2.5), diabetic 1.0 (0.5–2.0), p = 0.053; Fig 11] but not TRPV4 (not shown).

## Discussion

The TRP family appears to have differential expression in the peripheral nervous system, and specific changes in peripheral neuropathies.

### DRG

In this study we have described, to our knowledge for the first time, the presence of TRPV4 and TRPM8 in human sensory neurons. TRPV4 expression was not specific for any neuronal subtype in DRG and did not appear to be affected by injury in either DRG or peripheral nerves. The wide distribution of TRPV4 in both small and large neurons matches observations in mice [49]. The distribution of TRPM8 was confined to a small proportion of small/medium neurons. TRPM8 immunoreactive large calibre fibres with clear nodes of Ranvier were observed, suggesting labelling of Schwann cells-myelin in addition to fine weak fibres, as also observed by us in tooth pulp (unpublished observations). Small dot-like structures in the tissue surrounding the sensory neurons, apparent with antibodies in Figure 1 probably represents immunoreactivity in nerve fibres cut transverse to the plane of the sections. TRPM8 mRNA has been shown to be localised mainly in A- delta fibres/C-fibres in rat primary afferent neurones [47]. In rodents, TRPM8 and the cold activated receptor TRPA1 are also detected in sub-populations of small neurons. In mouse DRG, TRPM8 mRNA does not co-express with many of the classical markers of nociception including TRPV1[48]. However, TRPA1 is found in nociceptive sensory neurons in DRG and colocalises with TRPV1, CGRP and SP but not with TRPM8 in rat [9,47]. Co-expression of TRPA1 with TRPV1 could explain the paradoxical heat sensation which may be experienced on exposure to a very cold stimulus.

We have shown previously and confirmed in this study that TRPV1 and TRPV3 were present in human DRG neurons, mostly of small/medium diameter, and that the number of positive cells increased after DRG avulsion injury [4].

### Roots and Spinal Cord

There was a clear difference in the distribution of TRPV3 and TRPV1 in spinal nerve roots. TRPV3 was present in ventral but not dorsal roots, in contrast, TRPV1 was present in dorsal but negligible in ventral roots.

The strong immunoreactivity for TRPV3 in adult human motor neurons and ventral roots correlates well with studies in monkeys showing TRPV3 m-RNA in motor neurons and other neuronal elements [5]. Accordingly, we found strong TRPV3 immunoreactivity in motor neurons in adult rat (data not shown). The TRPV3-immunoreactive, large calibre fibres seen in control and acutely injured ventral spinal roots almost disappear after chronic injury. The weak TRPV3 immunoreactivity in dorsal roots is consistent with a preferential transport peripherally rather than centrally. It cannot be excluded that these observations reflect a difference in levels of TRPV3 protein or quality of the antibody.

TRPV1 immunoreactivity was not detected in ventral roots, which may be expected as it was not detected in motor neurons. In rats, TRPV1 has been shown to be transported from the DRG neurons both peripherally and centrally to laminae I and II of the spinal cord via the dorsal root [50]. In accord with this, we have shown that TRPV1 is present in spinal dorsal roots.

### Peripheral nerve-brachial plexus injury

In this study we have shown that both TRPV3 and TRPV1 are increased in peripheral nerve proximal to the site of injury in accord with our previous study of human DRG, [4]. The increase in injured nerves indicates that these receptors continue to be exported from the ganglion and/or accumulate in the nerve proximal to injury, despite overall reduced support from peripheral trophic factors e.g. NGF/GDNF. An increased availability of trophic factors to spared nerve fibres may be similar to the finding in an animal model of partial nerve injury. In this model it was suggested that undamaged fibres obtain more neurotrophin made available because of the reduced uptake by damaged fibres [20]. The increased TRPV receptors immunostaining seen in our study, in combination with the presence of regenerating fibres, may be particularly relevant for the development and persistence of pain. In addition, inflammatory mediators and neurotrophins derived from damaged Schwann cells or infiltrating macrophages at the injury site may contribute to enhance the production of vanilloid receptors [51-54].

The abundance of TRPV1 fibres in painful, hypersensitive skin relative to normal skin correlates well with the TRPV1 involvement in mechanisms of pain and hyperalgesia in a similar fashion to that described in injured nerve above. Corroborative evidence for the involvement of TRPV1-immunoreactive nerves in painful skin is shown in our recent work in vulvodynia and breast pain [38,55]. The lack of detection of TRPV3 immunoreactive fibres in skin may be similar to that described for dorsal roots.

### Peripheral neuropathic nerves

There was a clear decrease in the expression of TRPV1 in neuropathic nerves compared to controls. The cases were different, from distal axonal to demyelinating neuropathy. In all cases histology showed a decrease of nerve fibres and/or axonal atrophy.

### Skin and diabetic neuropathy

#### Keratinocytes

We have shown that TRPV3 is present in human keratinocytes, which is consistent with the report of TRPV3 in rodent keratinocytes [28]. Our report that TRPV1 and TRPV3 co-localize in human DRG suggested co-expression but we were unable to detect TRPV1 in human keratinocytes despite several reports describing TRPV1 in keratinocytes and other non-neuronal cells [29,30,56-59]. However these studies were performed in cultured keratinocytes and TRPV1 expression could therefore be a consequence of culture conditions. Alternatively, the lack of TRPV1 immunoreactivity in keratinocytes in the present study could reflect low level of expression or receptor conformational changes preventing antigen-antibody recognition.

Diabetic epidermis is known to be abnormally thin, so the trend we describe for decrease of TRPV3 in keratinocytes, may be related to changes of neurotrophins or other factors controlling skin differentiation and TRPV3 expression. Functional properties of vanilloid receptors in keratinocytes have been suggested to involve novel properties of transduction between the basal keratinocytes and sub-epidermal/epidermal nerve endings [28] or exocytosis of epidermal lamellar bodies, which is regulated by calcium influx [60]. A functional role for vanilloid receptors in keratinocytes remains unclear.

#### Innervation

In the present study, we have demonstrated that TRPV1 is decreased in both intra- and sub-epidermal fibres in diabetes resulting in the hypo-sensitivity typical of diabetic neuropathy. Similar results for intra-epidermal fibres have been shown in human diabetic skin using the pan-neuronal marker PGP-9.5 [39] whilst others have shown no change of TRPV1[40]. The observed decrease of TRPV1 may have been due simply to an overall reduction of innervation, a common feature of diabetes. To address this we used a neuronal marker for quantification of total innervation to normalise values. Ideally, antibodies to the pan-neuronal marker PGP-9.5 would have been used but these are not optimal on post-fixed tissue. Instead, comparisons were made using the nerve markers neurofilament and peripherin, which do not detect epidermal fibres, thus limiting our normalised analysis to sub-epidermal fibres. The density of TRPV1 sub-epidermal fibres was still clearly decreased in diabetic skin after normalisation with either of the above neuronal markers. In addition to a loss of fibres, our results therefore suggest that TRPV1 is down-regulated in remaining fibres.

## Conclusion

Vanilloid receptors are differentially regulated after nerve injury and in diabetic neuropathy. A change of expression and regulation of vanilloid receptors may play a role in sensory dysfunction, related to states of hyper/hypo-algesia. TRPV1 represents a more selective target than TRPV3 for pain, particularly for post-traumatic chronic hypersensitivity states.

## Competing interests

The author(s) declare that they have no competing interests.

## Authors' contributions

PF and MAC participated in immunohistology and drafted manuscript. GDS, CDB, IPC and CB provided antibodies, and participated in design of the study. RB and MS collected biopsies and participated to the coordination of the study. PA conceived the original study, its design and coordination. All authors read and approved the final manuscript.

## Pre-publication history

The pre-publication history for this paper can be accessed here:


